# Avitourism and Australian Important Bird and Biodiversity Areas

**DOI:** 10.1371/journal.pone.0144445

**Published:** 2015-12-23

**Authors:** Rochelle Steven, Clare Morrison, J. Michael Arthur, J. Guy Castley

**Affiliations:** 1 Environmental Futures Research Institute, Griffith School of Environment, Gold Coast campus, Griffith University, Gold Coast, Queensland, 4222, Australia; 2 Griffith School of Environment, Gold Coast campus, Griffith University, Gold Coast, Queensland, 4222, Australia; 3 Australian Rivers Institute, Griffith School of Environment, Griffith University, Gold Coast, Queensland, Australia; Centre for Cellular and Molecular Biology, INDIA

## Abstract

Formal protected areas will not provide adequate protection to conserve all biodiversity, and are not always designated using systematic or strategic criteria. Using a systematic process, the Important Bird and Biodiversity Area (IBA) network was designed to highlight areas of conservation significance for birds (i.e. IBA trigger species), and more recently general biodiversity. Land use activities that take place in IBAs are diverse, including consumptive and non-consumptive activities. Avitourism in Australia, generally a non-consumptive activity, is reliant on the IBA network and the birds IBAs aim to protect. However, companies tend not to mention IBAs in their marketing. Furthermore, avitourism, like other nature-based tourism has the potential to be both a threatening process as well as a conservation tool. We aimed to assess the current use of IBAs among Australian-based avitour companies’ marketing, giving some indication of which IBAs are visited by avitourists on organised tours. We reviewed online avitour itineraries, recorded sites featuring in descriptions of avitours and which IBA trigger species are used to sell those tours. Of the 209 avitours reviewed, Queensland is the most featured state (*n* = 59 tours), and 73% feature at least one IBA. Daintree (*n* = 22) and Bruny Island (*n* = 17) IBAs are the most popular, nationally. Trigger species represent 34% (*n* = 254 out of 747) of species used in avitour descriptions. The most popular trigger species’ are wetland species including; Brolga (*n* = 37), Black-necked Stork (*n* = 30) and Magpie Goose (*n* = 27). Opportunities exist to increase collaboration between avitour companies and IBA stakeholders. Our results can provide guidance for managing sustainability of the avitourism industry at sites that feature heavily in avitour descriptions and enhance potential cooperation between avitour companies, IBA stakeholders and bird conservation organisations.

## Introduction

While protected areas (PAs) have long been viewed as one of the most effective conservation measures, not all species are found within PA networks [1–3]. Birds, among many species, persist in a variety of areas including private reserves and other sites important for biodiversity, such as BirdLife International’s Important Bird and Biodiversity Area (IBA) network [4–6]. IBAs aim to increase awareness among governments and conservation practitioners of the importance of bird habitats worldwide. The IBA program was first established by the then International Council for Bird Preservation in 1989 with the production of a directory of IBAs for the European Union. There are currently more than 12,000 IBAs in more than 200 countries, with approximately half of the total IBA area captured within formal PAs [7]. IBAs are identified using a systematic selection process, largely based on the presence of various trigger species.

Trigger criteria are based on populations of species occurring in a defined area at certain population thresholds. The species may trigger one or more of the following criteria; globally threatened species (A1); range-restricted species (A2); biome-restricted species (A3); congregations (A4) [8]. More detailed descriptions of these criteria are available in Supporting Information (S1 Table). Some species may trigger more than one criterion; for example, the Gouldian Finch, which has triggered IBAs under both A1 and A3 categories [9]. Despite targeting bird species and biodiversity declines, IBAs (protected and unprotected) are subject to a mix of human activities and land uses, including; selective logging or forestry, small scale agriculture, and nature-based tourism and recreation [10].

Avitourism is a growing niche sector of the broader nature-based tourism industry with avitourists travelling great distances to see bird species they may not have seen before or have the opportunity to see regularly near their place of residence [11–14]. Avitourists often acquire information about desired species, destinations and tours prior to travel, including via; magazines, birding forums, online blogs and tour company websites [15–19]. While some avitourists travel independently, many engage the services of a commercial company, which often ensures visits to sites with high species richness or maximises their likelihood of seeing particular species [17]. Despite the fact that IBAs are designated based on the presence of birds that would appeal to avitourists [12, 13, 17, 18], we do not have an adequate understanding of how these areas are targeted by the avitourism industry. In light of such new information, conservation practitioners in IBAs can then put strategies in place to maximise the potential benefit of avitourism activities [20, 21], while also managing the possible negative impacts of avitourism [12, 18, 22–25].

The avifauna of Australia features high levels of endemism and is sought after by many avitourists from the Northern Hemisphere [26]. In the United Kingdom and United States of America (where most international avitourism source markets are located) the bird assemblages are very different to those in Australia. In Australia, the IBA network comprises over 300 sites, approximately half of which overlap with the legislated PA network [27]. As an emerging geographic area of research into avitourism we begin to explore the relationship between avitour companies and IBAs in Australia via a content analysis of Australian avitour company websites [28–30]. Only Australian companies were the focus of this study to assess the relationship between domestic avitour companies and the IBA network in Australia. While international avitour companies visit numerous countries and regions, the Australian companies reviewed here tend to focus predominantly on Australian destinations. Due to their reliance on Australian birds it is in their interests to ensure birds are conserved in the wild and this study attempts to examine this relationship further.

Avitourism companies commonly use detailed descriptions to communicate the sites they visit on pre-organised avitours and the types of birds potential avitourists are likely to see on those tours. These data are freely available online, and research approaches such as this are becoming popular and cost effective methods to assess various nature-based tourism and recreation trends [28–30]. We formulated an attractiveness index of the Australian IBAs to test whether the IBAs that receive the most attention on avitour descriptions exhibit the attributes (i.e. high species richness, large number of each trigger species, and greater proximity) one would expect to be attractive to potential avitourists [12, 13, 18, 19]. These attributes may also be useful for managers of IBAs to identify opportunities for avitourism activities or correspondingly where some activities may need to be restricted. Specifically, we address the following questions: 1) Which Australian regions do avitours feature most frequently in their online marketing? 2) What are the characteristics that make Australian IBAs popular for avitours? and 3) What role do trigger species play in the marketing of avitours in online itinerary descriptions? Our results will provide some insight into the existing and potential ways avitourism can enhance conservation in IBAs.

## Methods

### Data Collection

We used a content analysis based approach [28–30]. Internet searches for avitours in Australia were undertaken from January 2013 to April 2013, to determine how local avitour companies (which provide services to both domestic and international avitourists) are utilising the Australian bird species and habitats to sell avitourism. The information obtained is freely available online to anyone seeking information on avitours run by Australian avitour companies inside Australia, and required no direct participation of the companies in the study. The avitour companies also retain anonymity in the study.

Each company usually provides a list of the tours they offer. These include tours that are fixed date tours or are delivered upon inquiry by the tourists. Each tour may run from as little as a day or two up to a month long with most tours approximately one week long. The tours reviewed here were generally advertised for the 2013/2014 period. After visiting the avitour websites in the subsequent year it appears the avitours offered may change slightly from year to year; hence a reproduction of this study in another year may produce different results. The key patterns and outcomes however, are likely to remain similar.

The locations featured were checked to determine whether they overlap or are contained within the IBA network, thereby providing an indication of IBA visitation for the purposes of avitourism. In some cases it is possible that IBAs are visited during tours, but insufficient details prevents confirmation. Consequently, the levels of visitation to IBAs may be an underestimate. We also collected data pertaining to the species (i.e. species name, common name, family, conservation status, and IBA trigger criteria) mentioned in the descriptions of avitours.

### Data Analysis

To examine broad geographic patterns of preference for IBAs across the Australian states (research question 1) we conducted goodness of fit tests, where tours that visit more than one state gave counts for all states featured (Lord Howe Island was included in New South Wales, and Christmas Island in Western Australia). For IBA preference analyses (research question 2), we calculated an attractiveness index using a combination of site and bird assemblage attributes (species richness, number of trigger [A1, A2, A3 A4i/A4ii] species and distance to nearest town or city) (Table 1). Each attribute could receive a score of 1 (least attractive) to 4 (most attractive), which were subsequently averaged for each IBA to produce a score for overall attractiveness (Table 1) [28]. We analysed the attractiveness index data via logistical analysis (generalised linear model—binomial distribution, logit link) to examine differences between the attractiveness index of IBAs with and without avitours (binary dependent variable) in the program SPSS (Version 22). We subsequently tested the data for IBAs with tours (non-zero values) using linear mixed modelling (random intercept, random slope) to assess the effect of increasing attractiveness on the number of avitours that feature IBAs in their itineraries. The data for IBAs with avitours were transformed using a natural log transformation, due to heterogeneity of variance. For the trigger species analysis, many species can meet the criteria for more than one trigger category. As a result, due to assumptions of independence for most statistical tests, specific trigger species used in avitour itineraries were examined descriptively.

**Table 1:** IBA Attractiveness score metrics.

IBA attribute	Attractiveness Score			
	1	2	3	4
Species richness	<100spp.	100-200spp.	200-300spp.	>300spp.
A1 Threatened Species	Zero spp.	1–2 spp.	3–4 spp.	>4 spp.
A2 Restricted-range Species	Zero spp.	1–3 spp.	4–5 spp.	>5 spp.
A3 Biome-restricted Species	Zero spp.	1–5 spp.	6–10 spp.	>10 spp.
A4i/ii Congregatory Species	Zero spp.	Waterbirds A4i	Seabirds A4ii	Waterbirds and seabirds
Distance to town/city	>500 km	201–500 km	100km-200 km	<100 km

Spp. = species

For all statistical analyses, alpha was set at 0.05. Standard error for all quoted means is represented by a ± symbol.

## Results

### Geography of avitours in Australian IBAs

Of the 209 avitours included in this analysis (run by 34 companies), 153 (73%) feature at least one IBA in their tour itineraries. In general, avitour companies do not mention the fact that the sites they visit are IBAs (i.e. 32 of the 34 companies reviewed). These 209 tours represented 97% of all tours retrieved from internet searches, with the remaining 3% not including details about sites visited. An average of 2.36 (±0.163) IBAs are visited per tour. Of the 310 IBAs in Australia, 100 are featured by avitour companies in their itineraries (S2 Table). This is likely to be an underestimate given the reliance on detailed information about destinations on company webpages. Some companies specialise in one regional area and others lead tours Australia-wide. There is a significant difference among the states for IBAs featured most often in avitour itinerary descriptions (*χ* ^*2*^ = 27.0, *df* = 6, *p* <0.001). Queensland is the top destination among domestic avitour companies in Australia with 59 tours and the most commonly featured IBA is Daintree (*n* = 22) equating to 10% of all avitours. Victoria features in 35 tours, and New South Wales in 33 tours (Table 2). The Northern Territory has almost the same number of companies as Queensland (14 and 13, respectively), but the Northern Territory companies offer fewer tours than those in Queensland (31 and 59, respectively). Other IBAs that feature prominently in itineraries are; Bruny Island, near Tasmania (*n* = 17), Adelaide and Mary River Floodplains and Wooroonooran IBAs (*n* = 16, each) and the Atherton Tablelands IBA (*n* = 15). Fifty-six IBAs (36.6% of the 153) attract only one or two tours (S2 Table).

**Table 2:** Geographical spread of avitours in Australian states.

State	Number of avitours	Number of companies	Number of IBAs per state
Northern Territory	31	14	31
Queensland	59	13	53
Victoria	35	12	37
New South Wales (incl. Lord Howe Island)	33	9	45
South Australia	28	9	38
Tasmania	20	9	43
Western Australia (incl. Ashmore Reef, Christmas Island)	28	9	75

One tour may visit multiple states; therefore these numbers do not cumulate to the total number of tours reviewed

### IBA attractiveness analysis

**Fig 1:**
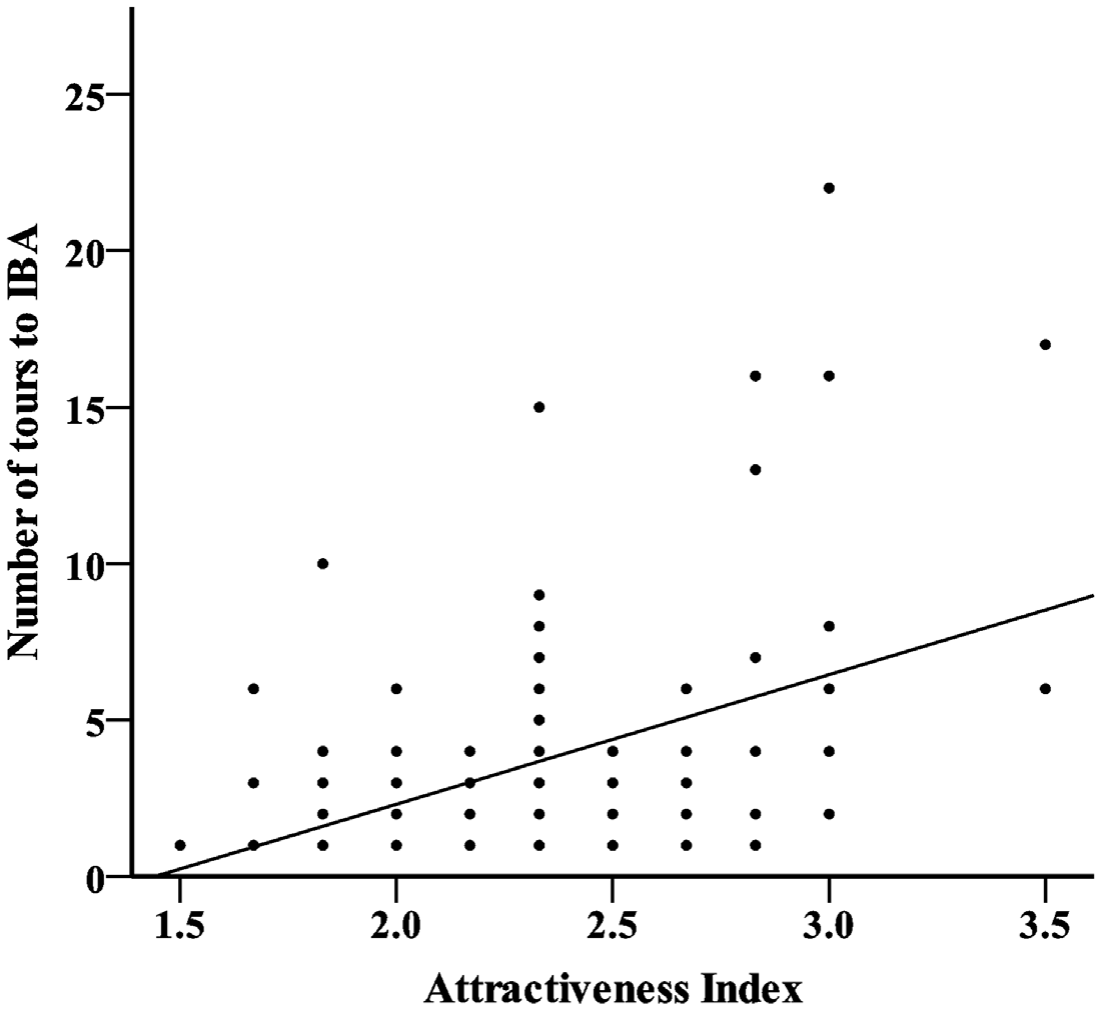
Relationship of attractiveness of Australian IBAs and the number of avitours they feature in.

The mean IBA attractiveness score across all Australian IBAs was 2.119 ±0.024) (range 1.167–3.5). The state with the highest attractiveness score for IBAs was New South Wales ($\bar{x}$ = 2.244) and the lowest was Northern Territory ($\bar{x}$ = 1.984) (S2 Table). Our analysis found that there is a significant difference in attractiveness of IBAs with and without mention in avitour itineraries where, for each unit increase in attractiveness score, the odds of that IBA featuring in avitour itineraries increases by six fold (*χ* ^*2*^ = 33.2, *df* = 1, *p* <0.001). Furthermore, for IBAs that do feature in itineraries (non-zero values), the higher the attractiveness score the more avitours the IBAs appeal to, with each unit increase in attractiveness score resulting in an increase of 2.4 tours (F = 19.1, *df* = 1,90, *p* <0.001) (Fig 1). The state where the IBA is located appears to have only a marginal effect on this result, explaining only 1% of the variation in the best model.

### Species in avitour descriptions

A total of 196 tours conducted by 34 companies describe the species an avitourist might expect to see on each of the tours. The mean number of species mentioned in avitour descriptions (for tours that gave species lists) was 34 (±2.87) species. Of the 747 species used across all avitour descriptions, 254 are trigger species for at least one IBA in Australia (including Christmas and Lord Howe Island IBAs). While each species may meet the criteria for more than one IBA trigger category, the number of species used in avitour descriptions for each of those categories was as follows; A1 –*n* = 59; A2 –*n* = 66; A3 –*n* = 108; A4i –*n* = 80; A4ii–*n* = 32. The average number of tours for species in each trigger category are; A1−$\bar{x}$ = 9.53; A2−$\bar{x}$ = 8.70; A3−$\bar{x}$ = 9.49; A4i−$\bar{x}$ = 11.34; A4ii−$\bar{x}$ = 5.88.

For all trigger categories combined the top three species mentioned in avitour descriptions were A4i species; Brolga (*n* = 37) (19% of all reviewed) followed by Black-necked Stork (*n* = 30) and Magpie Goose (*n* = 27) (Table 3). Among the other trigger categories, globally threatened (A1) species used most often in avitour descriptions included the Black-necked Stork (*n* = 30), followed by Gouldian Finch (Near Threatened, *n* = 26) and Malleefowl (Vulnerable, *n* = 22). Rainbow Pitta was the A2 species mentioned most often (*n* = 18) followed by Forty-spotted Pardalote (*n* = 17). The latter species, a Tasmanian endemic, also triggered criteria A1 and A3. The Gouldian Finch was the top A3 species featured in avitour descriptions (*n* = 26). The Regent Parrot also featured often in avitour descriptions (*n* = 24) along with the Black Honeyeater (*n* = 22). The most frequently mentioned A4ii species were Little Penguin (*n* = 24) and Rainbow Bee-eater (*n* = 19). Species that were mentioned least often are triggers for IBAs that are located off of the Australian mainland and thus attract fewer avitours (e.g. Sub-Antarctic penguins, Christmas Island species).

**Table 3:** Top 20 trigger species per IBA trigger category in avitour descriptions.

Common Name	Scientific Name	A1	A2	A3	A4i	A4ii	# tours
Brolga	*Grus rubicunda*				1		37
Black-necked Stork (Jabiru)	*Ephippiorhynchus asiaticus*	1			1		30
Magpie Goose	*Anseranas semipalmata*				1		27
Gouldian Finch	*Erythrura gouldiae*	1		1			26
Little Penguin	*Eudyptula minor*					1	24
Regent Parrot	*Polytelis anthopeplus*			1			24
Black Honeyeater	*Certhionyx niger*			1			22
Malleefowl	*Leipoa ocellata*	1					22
Red Goshawk	*Erythrotriorchis radiatus*	1					22
Red-necked Avocet	*Recurvirostra novaehollandiae*				1		22
Australian Bustard	*Ardeotis australis*	1					21
Australian Pelican	*Pelecanus conspicillatus*				1		21
Bush Stone-curlew	*Burhinus grallarius*	1					21
Diamond Firetail	*Stagonopleura guttata*	1					21
Freckled Duck	*Stictonetta naevosa*				1		21
Flame Robin	*Petroica phoenicea*	1					20
Masked Finch	*Poephila personata*			1			20
Green Pygmy-Goose	*Nettapus pulchellus*				1		19
Pink Robin	*Petroica rodinogaster*			1			19
Pink-eared Duck	*Malacorhynchus membranaceus*				1		19
Rainbow Bee-eater	*Merops ornatus*					1	19
Royal Spoonbill	*Platalea regia*				1		19
Pied Honeyeater	*Certhionyx variegatus*			1			18
Purple-gaped Honeyeater	*Lichenostomus cratitius*			1			18
Rainbow pitta	*Pitta iris*		1	1			18
Banded Stilt	*Cladorhynchus leucocephalus*				1		17
Comb-crested Jacana	*Irediparra gallinacea*				1		17
Forty-spotted Pardalote	*Pardalotus quadragintus*	1	1	1			17
Musk Duck	*Biziura lobata*				1		17
Black-fronted Dotterel	*Elseyornis melanops*				1		16
Black-throated Finch	*Poephila cincta*	1					16
Golden Bowerbird	*Prionodura newtoniana*		1	1			16
Hooded Plover	*Thinornis rubricollis*	1					16
Short-tailed Shearwater	*Puffinus tenuirostris*					1	16
Australian Pratincole	*Stiltia isabella*				1		15
Grey Teal	*Anas gracilis*				1		15
Inland Dotterel	*Charadrius australis*			1			15
Red-kneed Dotterel	*Erythrogonys cinctus*				1		15
Victoria’s Riflebird	*Ptiloris victoriae*		1	1			15
Wandering Whistling-duck	*Dendrocygna arcuata*				1		15
Blue-billed Duck	*Oxyura australis*	1			1		14
Cinnamon Quail-thrush	*Cinclosoma cinnamomeum*			1			14
Hooded Parrot	*Psephotus dissimilis*		1	1			14
Long-tailed Finch	*Poephila acuticauda*			1			14
Northern Rosella	*Platycercus venustus*			1			14
Rock Parrot	*Neophema petrophila*			1			14
Sandstone Shrike-thrush	*Colluricincla woodwardi*			1			14
White-lined Honeyeater	*Meliphaga albilineata*		1	1			14
Black-eared Miner	*Manorina melanotis*	1		1			13
Black-headed Honeyeater	*Melithreptus affinis*		1	1			13
Chestnut-quilled Rock-Pigeon	*Petrophassa rufipennis*		1	1			13
Star Finch	*Neochmia ruficauda*	1					13
Strong-billed Honeyeater	*Melithreptus validirostris*		1	1			13
Swift Parrot	*Lathamus discolor*	1					13
Tooth-billed Bowerbird	*Scenopoeetes dentirostris*		1	1			13
Western Whipbird	*Psophodes nigrogularis*	1	1	1			13
Yellow-throated Honeyeater	*Lichenostomus flavicollis*		1	1			13
Atherton Scrubwren	*Sericornis keri*		1	1			12
Beach Stone-Curlew	*Esacus giganteus*	1					12
Chestnut Rail	*Eulabeornis castaneoventris*		1				12
Golden-shouldered Parrot	*Psephotus chrysopterygius*	1	1	1			12
Red-lored Whistler	*Pachycephala rufogularis*	1	1	1			12
Sarus Crane	*Grus antigone*	1					12
Scrubtit	*Acanthornis magna*		1	1			12
Tasmanian Native-hen	*Gallinula mortierii*		1	1			12
Chestnut-breasted Whiteface	*Aphelocephala pectoralis*	1	1	1			11
Mallee Emu-wren	*Stipiturus mallee*	1	1	1			11
Yellow-faced Honeyeater	*Lichenostomus chrysops*					1	10
Brown Booby	*Sula leucogaster*					1	9
Shy Albatross	*Thalassarche cauta*					1	9
Spangled Drongo	*Dicrurus bracteatus*					1	9
Australian Gannet	*Morus serrator*					1	8
Lesser Frigatebird	*Fregata ariel*					1	8
Double-banded Plover	*Charadrius bicinctus*					1	7
Greater Frigatebird	*Fregata minor*					1	7
Flesh-footed Shearwater	*Puffinus carneipes*					1	6
Masked Booby	*Sula dactylatra*					1	6
Wedge-tailed Shearwater	*Puffinus pacificus*					1	6
Flock Bronzewing	*Phaps histrionica*					1	5
Great-winged Petrel	*Pterodroma macroptera*					1	5
Red-footed Booby	*Sula sula*					1	4
Red-tailed Tropicbird	*Phaethon rubricauda*					1	4
Fairy Prion	*Pachyptila turtur*					1	3
Providence Petrel	*Pterodroma solandri*					1	3

This is not a complete list of all trigger species used in avitour descriptions. Only the top 20 for each trigger category could be displayed.

## Discussion

### The connection between avitourism and Australian IBAs

The IBA network clearly plays an important role in the avitourism industry in Australia, with almost three quarters of avitours reviewed in our study featuring at least one IBA. Featured sites include those that have conservation significant species present (i.e trigger species) and high species richness. This represents an interesting opportunity and also a challenge for conservation managers in IBAs, where balancing the potential positive and negative effects of tourism needs to be considered. There is potential to raise awareness of the conservation status of these birds among avitour operators and their clients, given they appear to be drivers of preference among avitour companies choice of destinations. Conversely, the challenge lies in managing the potential impacts avitourism can have on birds and their habitats [22–25], where certain species and sites may be over-used by avitour companies in order to deliver the right experience to paying avitourists. Our study has demonstrated that threatened species feature prominently in avitour companies’ marketing, with almost half of the top ten trigger species listed as globally threatened (e.g. Black-necked Stork, Gouldian Finch, Malleefowl etc.). Landholders and managers of sites where these species are present, which are relied upon by avitour companies, need to minimise potential disturbance to these birds, especially during breeding seasons and other times of vulnerability [18, 24, 25]. Engagement and education among key stakeholders (i.e. BirdLife partners, avitour companies, PA government agencies etc.) will be necessary to manage these outcomes. Despite the opportunity avitour companies have to incorporate information about IBAs into their products, the IBA network is largely overlooked within the marketing and communication about sites featured by avitour companies. If avitourism is to fulfil its reputation as a sustainable form of nature-based tourism [31], then strengthening the connection among avitour companies, avitourists and IBAs would be a mutually beneficial approach. The success of these mutualisms is of course dependent on a number of factors captured within the broader definitions of ecotourism [32] and sustainable tourism [32, 33]. These include the long-term investment (financial and social capital) in these ventures, investment and economic benefit to local communities, development of pro-conservation behaviours [34]. If avitourists were actively informed about the IBA program, they may be more likely to show either monetary or political support for IBAs they visit. Similarly, if the avitourists are attracted to IBAs as key birding sites, avitour companies may use this as a marketing strategy and incorporate it into how they describe avitour itineraries. This apparent disconnect could be attributable to the Australian IBA’s embryonic status for, the network having been established as recently as 2009 [27].

### Future directions in research

Our findings add to an important but limited body of knowledge examining IBA monitoring and their effectiveness at conserving birds and biodiversity [35–37]. Few studies have investigated land use activities within IBAs, and here we have explored this in the context of tourism focussed on birds. There are, however, several aspects that are worthy of further discussion. Firstly, it is unclear what the likelihood of seeing a species advertised on an avitour itinerary is during an organised tour. Previous research has used a visibility index to predict the likelihood a species would be seen during a tourism experience using various physical and behavioural attributes [28]. This approach gives an indication of whether the species being used to entice avitourists to take a particular tour are likely to be observed by the tourists. This does not, however, take into account the methods used by an individual tour guide in the field to entice the birds to make themselves known to observers, such as the use of call playback.

Another avenue requiring further examination is related to site accessibility and tourist infrastructure for enhancing avitourism across the IBA network in Australia. While we incorporated a distance metric into our attractiveness score, this was fairly rudimentary in nature. Logistical aspects are frequently cited as key challenges to avitourism in remote areas, along with socio-political instability in some countries [13, 17, 38–40]. More in-depth analyses might consider land tenure (and therefore public access to IBAs), road and other transport networks, and IBAs with multiple entry points. While remote areas may be limited in their ability to benefit from avitourism economically, it also means the natural environment is less likely to be subjected to the potentially damaging effects of tourism [12, 22–25, 41, 42]. With approximately one third of the IBA network currently featured in avitour companies’ itineraries, widespread tourism threats are unlikely. However, there are opportunities for increased cooperation and communication among key stakeholders [43–45]. Increased public attention and patronage by avitourists may also bring additional political support for the IBA program in the form of formal protection and funding for management at some sites [46].

In conclusion, this study aimed to examine the role the IBA network and its corresponding trigger species play in the way avitour companies sell Australian birding to potential visitors. While monitoring and management of the IBA program continues to be a challenge globally [10], exploring novel approaches to increase awareness about the program are also important. We have demonstrated the importance of the IBA network to the avitourism industry in Australia. This study could readily be replicated for other countries to assess the current role of IBAs in avitourism elsewhere (e.g. South Africa). Ecologically significant trigger species play a key role in the marketing of avitourism in Australia. However, the general findings here are not necessarily limited to Australian IBAs and have addressed gaps in linkages between science and tourism research raised previously [45]. Furthermore, decisions about how best to manage tourism with respect to IBAs and significant species should be based on all of the available information, including that presented here.

## Supporting Information

S1 Table Definitions of trigger species criteria used in the identification of IBAs in Australia. (DOCX)

S2 Table Avitours to IBAs and metrics used to calculate attractiveness score. (DOCX)
