# Supplementary material for: Avitourism and Australian Important Bird and Biodiversity Areas
Source: PLoS One. 2015 Dec 23;10(12):e0144445. doi: 10.1371/journal.pone.0144445 (PMC4689425; doi:10.1371/journal.pone.0144445)
Supplement: S1 Table — (DOCX) [file pone.0144445.s001.docx]

**S1 Table.** **Definitions of trigger species criteria used in the identification of IBAs in Australia.**

Definitions are given for criteria that were relevant to the IBAs and trigger species concerned for this study. Criteria not included are A4iii – sites holding >20,000 pairs of waterbirds or >10,000 pairs of seabirds, and A4iv – sites known to exceed thresholds for migratory birds at bottleneck sites. There are no IBAs in Australia that were identified based on either of these trigger criteria.

| Criteria code | Definition |
| --- | --- |
| A1 | Globally threatened species  Criterion: The site is known or thought regularly to hold significant numbers of a globally threatened species, or other species of global conservation concern. Population threshold dependent. |
| A2 | Restricted-range species  Criterion: The site is known or thought to hold a significant component of a group of species whose breeding distributions define an Endemic Bird Area or Secondary Area. |
| A3 | Biome-restricted species  Criterion: The site is known or thought to hold a significant component of the group of species whose distributions are largely or wholly confined to one biome. |
| A4i | Congregations  Criterion: Site known or thought to hold, on a regular basis, > 1% of a biogeographic population of a congregatory waterbird species. |
| A4ii | Congregations  Criterion: Site known or thought to hold, on a regular basis, > 1% of the global population of a congregatory seabird or terrestrial species. |

Source: BirdLife International 2014b
