# Supplementary material for: Avitourism and Australian Important Bird and Biodiversity Areas
Source: PLoS One. 2015 Dec 23;10(12):e0144445. doi: 10.1371/journal.pone.0144445 (PMC4689425; doi:10.1371/journal.pone.0144445)
Supplement: S2 Table — (DOCX) [file pone.0144445.s002.docx]

**S2 Table. Avitours to IBAs and metrics used to calculate attractiveness score.**

| IBA | # Tours | Species Richness | Species Rich Score | A1 Score | A2 Score | A3 Score | A4 (i/ii) Score | Distance Score | Attractiveness Score |
| --- | --- | --- | --- | --- | --- | --- | --- | --- | --- |
| Adelaide & Mary River Floodplains | 16 | 269 | 3 | 2 | 2 | 4 | 2 | 4 | 2.833 |
| Adele Island | 1 | 91 | 1 | 1 | 1 | 1 | 4 | 2 | 1.667 |
| Albatross Island & Black Pyramid Rock |  | 154 | 2 | 2 | 1 | 1 | 3 | 4 | 2.167 |
| Alligator Rivers Floodplains |  | 278 | 3 | 3 | 2 | 4 | 2 | 3 | 2.833 |
| Anderson Inlet |  | 296 | 3 | 2 | 1 | 1 | 2 | 4 | 2.167 |
| Anson Bay, Daly & Reynolds River Floodplains |  | 247 | 3 | 2 | 2 | 3 | 2 | 3 | 2.500 |
| Arafura Swamp |  | 230 | 3 | 2 | 1 | 1 | 2 | 2 | 1.833 |
| Araluen-Wungong | 2 | 212 | 3 | 2 | 3 | 4 | 1 | 4 | 2.833 |
| Arnhem Plateau | 4 | 263 | 3 | 2 | 3 | 4 | 1 | 2 | 2.500 |
| Ashmore Reef | 1 | 13 | 1 | 1 | 1 | 1 | 4 | 1 | 1.500 |
| Atherton Tablelands | 15 | 318 | 4 | 2 | 1 | 1 | 2 | 4 | 2.333 |
| Australian Alps | 4 | 294 | 3 | 2 | 2 | 1 | 1 | 2 | 1.833 |
| Babel Island Group |  | 164 | 2 | 1 | 1 | 1 | 4 | 4 | 2.167 |
| Barmah-Millewa | 2 | 271 | 3 | 3 | 2 | 1 | 1 | 4 | 2.333 |
| Barrington Tops & Gloucester Tops | 2 | 318 | 4 | 2 | 3 | 2 | 1 | 3 | 2.500 |
| Barrow Island |  | 94 | 1 | 2 | 1 | 2 | 2 | 2 | 1.667 |
| Bedout Island |  | 15 | 1 | 1 | 1 | 1 | 3 | 4 | 1.833 |
| Bellarine Wetlands |  | 303 | 4 | 2 | 1 | 1 | 2 | 4 | 2.333 |
| Ben Lomond |  | 158 | 2 | 2 | 4 | 4 | 1 | 4 | 2.833 |
| Bendigo Box-Ironbark Region | 3 | 266 | 3 | 3 | 1 | 1 | 1 | 3 | 2.000 |
| Benger Swamp |  | 189 | 2 | 2 | 1 | 1 | 1 | 3 | 1.667 |
| Betsey Island |  | 162 | 2 | 1 | 1 | 1 | 4 | 4 | 2.167 |
| Billiatt | 1 | 245 | 3 | 2 | 2 | 2 | 1 | 3 | 2.167 |
| Bindoon-Julimar |  | 210 | 3 | 2 | 1 | 3 | 1 | 4 | 2.333 |
| Binya & Cocoparra | 1 | 240 | 3 | 2 | 1 | 1 | 1 | 3 | 1.833 |
| Blue Mud Bay |  | 214 | 3 | 1 | 1 | 1 | 2 | 1 | 1.500 |
| Booby Island (Kimberley) |  | 13 | 1 | 1 | 1 | 1 | 3 | 2 | 1.500 |
| Boodjamulla | 2 | 208 | 3 | 3 | 2 | 4 | 1 | 2 | 2.500 |
| Boolcoomatta, Bindarrah & Kalkaroo Stations |  | 180 | 2 | 2 | 1 | 1 | 1 | 2 | 1.500 |
| Bountiful Islands |  | 92 | 1 | 1 | 1 | 1 | 2 | 2 | 1.333 |
| Boxen Island & Big Black Reef |  | 46 | 1 | 1 | 1 | 1 | 2 | 3 | 1.500 |
| Brisbane Water |  | 353 | 4 | 3 | 1 | 1 | 1 | 4 | 2.333 |
| Broad Sound |  | 321 | 4 | 2 | 2 | 1 | 2 | 3 | 2.333 |
| Brook Islands |  | 20 | 1 | 1 | 1 | 1 | 4 | 3 | 1.833 |
| Bruny Island | 17 | 172 | 2 | 3 | 4 | 4 | 4 | 4 | 3.500 |
| Buckingham Bay |  | 207 | 3 | 1 | 1 | 1 | 2 | 1 | 1.500 |
| Buckley River | 1 | 220 | 3 | 2 | 2 | 3 | 1 | 4 | 2.500 |
| Budderoo & Barren Grounds | 1 | 299 | 3 | 2 | 2 | 2 | 1 | 4 | 2.333 |
| Bulgunnia |  | 170 | 2 | 2 | 2 | 3 | 1 | 3 | 2.167 |
| Bulloo Floodplain | 2 | 211 | 3 | 1 | 2 | 2 | 2 | 2 | 2.000 |
| Bundarra-Barraba |  | 270 | 3 | 2 | 3 | 2 | 1 | 4 | 2.500 |
| Bunya Mountains & Yarraman |  | 306 | 4 | 2 | 3 | 2 | 1 | 3 | 2.500 |
| Busselton Wetlands |  | 223 | 3 | 1 | 1 | 1 | 2 | 4 | 2.000 |
| Cabbage Tree & Boondelbah Islands |  | 49 | 1 | 2 | 1 | 1 | 3 | 4 | 2.000 |
| Cadell & Blyth Floodplains | 1 | 226 | 3 | 1 | 1 | 1 | 2 | 2 | 1.667 |
| Calingiri |  | 194 | 2 | 2 | 2 | 2 | 1 | 3 | 2.000 |
| Cape Portland |  | 210 | 3 | 2 | 3 | 3 | 2 | 3 | 2.667 |
| Cape York to Cape Grenville Islands |  | 30 | 1 | 1 | 1 | 1 | 4 | 3 | 1.833 |
| Capertee Valley | 4 | 285 | 3 | 3 | 2 | 2 | 1 | 3 | 2.333 |
| Capricornia Cays |  | 210 | 3 | 1 | 1 | 1 | 3 | 3 | 2.000 |
| Carnac Island (Perth) |  | 36 | 1 | 2 | 1 | 1 | 2 | 4 | 1.833 |
| Carrum Wetlands |  | 264 | 3 | 2 | 1 | 1 | 2 | 4 | 2.167 |
| Cataby |  | 200 | 3 | 2 | 2 | 2 | 1 | 3 | 2.167 |
| Central Flinders Island |  | 139 | 2 | 2 | 4 | 3 | 1 | 3 | 2.500 |
| Central NSW Mallee | 1 | 237 | 3 | 2 | 2 | 2 | 1 | 3 | 2.167 |
| Chalky, Big Green and Badger Island Groups |  | 159 | 2 | 2 | 1 | 1 | 4 | 3 | 2.167 |
| Cheetham & Altona |  | 303 | 4 | 1 | 1 | 1 | 2 | 4 | 2.167 |
| Christmas Island | 1 | 47 | 1 | 4 | 1 | 1 | 3 | 1 | 1.833 |
| Clarke Range |  | 305 | 4 | 2 | 2 | 2 | 1 | 4 | 2.500 |
| Coastal Wet Tropics | 6 | 357 | 4 | 2 | 3 | 2 | 1 | 4 | 2.667 |
| Coffin Bay | 1 | 232 | 3 | 3 | 2 | 2 | 2 | 2 | 2.333 |
| Conondale Range |  | 327 | 3 | 2 | 2 | 2 | 1 | 3 | 2.167 |
| Cooloola & Fraser Coast |  | 364 | 4 | 3 | 3 | 2 | 1 | 3 | 2.667 |
| Coomallo |  | 198 | 2 | 2 | 2 | 3 | 1 | 3 | 2.167 |
| Coongie Lakes |  | 170 | 2 | 2 | 2 | 3 | 2 | 4 | 2.500 |
| Cooper Floodplain Below Windorah |  | 187 | 2 | 1 | 1 | 1 | 2 | 2 | 1.500 |
| Coorong | 2 | 310 | 4 | 3 | 2 | 2 | 2 | 3 | 2.667 |
| Coringa-Herald Reefs |  | 44 | 1 | 1 | 1 | 1 | 4 | 2 | 1.667 |
| Corner Inlet |  | 293 | 3 | 3 | 1 | 1 | 2 | 4 | 2.333 |
| Cradle Mountain | 2 | 153 | 2 | 2 | 4 | 4 | 1 | 4 | 2.833 |
| Curtis Island |  | 159 | 2 | 1 | 1 | 1 | 3 | 4 | 2.000 |
| Daintree | 22 | 348 | 4 | 2 | 4 | 4 | 1 | 3 | 3.000 |
| Dampier Saltworks |  | 175 | 2 | 1 | 2 | 1 | 2 | 4 | 2.000 |
| Devilbend Reservoir |  | 255 | 3 | 2 | 1 | 1 | 2 | 4 | 2.167 |
| Diamantina & Astrebla Grasslands | 1 | 189 | 2 | 3 | 1 | 3 | 2 | 3 | 2.333 |
| Diamantina Floodplain | 1 | 178 | 2 | 2 | 2 | 2 | 2 | 2 | 2.000 |
| Discovery Bay to Piccaninnie Ponds | 1 | 299 | 3 | 3 | 2 | 2 | 1 | 4 | 2.500 |
| Douglas-Apsley |  | 158 | 2 | 2 | 4 | 4 | 1 | 4 | 2.833 |
| Dragon Rocks | 1 | 170 | 2 | 2 | 1 | 2 | 1 | 2 | 1.667 |
| Dunn Rock & Lake King |  | 188 | 2 | 2 | 1 | 3 | 1 | 2 | 1.833 |
| East Borden |  | 193 | 2 | 2 | 1 | 2 | 1 | 3 | 1.833 |
| Eastern Flinders Island |  | 169 | 2 | 2 | 1 | 1 | 2 | 3 | 1.833 |
| Eclipse Island (Albany) |  | 162 | 2 | 1 | 1 | 1 | 3 | 4 | 2.000 |
| Egg Island (Bass Strait) |  | 46 | 1 | 1 | 1 | 1 | 2 | 4 | 1.667 |
| Egg Islands (Huon Estuary) |  | 196 | 2 | 2 | 1 | 1 | 1 | 4 | 1.833 |
| Eighty Mile Beach |  | 217 | 3 | 1 | 2 | 1 | 2 | 2 | 1.833 |
| Exmouth Gulf Mangroves |  | 194 | 2 | 1 | 2 | 1 | 2 | 2 | 1.667 |
| Faure & Pelican Islands (Shark Bay) |  | 121 | 2 | 2 | 1 | 1 | 2 | 1 | 1.500 |
| Fitzgerald River | 1 | 245 | 3 | 3 | 2 | 4 | 1 | 3 | 2.667 |
| Fitzroy Floodplain & Delta |  | 336 | 4 | 1 | 2 | 1 | 2 | 4 | 2.333 |
| Fivebough & Tuckerbil Swamps |  | 238 | 3 | 2 | 1 | 1 | 2 | 4 | 2.167 |
| Flinders Ranges | 10 | 198 | 2 | 1 | 2 | 2 | 1 | 3 | 1.833 |
| Fog Bay & Finniss River Floodplains |  | 257 | 3 | 1 | 1 | 1 | 2 | 4 | 2.000 |
| Forsyth, Passage & Gull Islands |  | 151 | 2 | 1 | 1 | 1 | 4 | 2 | 1.833 |
| Fortescue Marshes |  | 154 | 2 | 3 | 1 | 3 | 2 | 2 | 2.167 |
| Franklin Sound Islands |  | 159 | 2 | 2 | 1 | 1 | 4 | 3 | 2.167 |
| Gabo & Tullaberga Islands |  | 225 | 3 | 1 | 1 | 1 | 3 | 4 | 2.167 |
| Gammon Ranges and Arkaroola | 1 | 183 | 2 | 1 | 2 | 2 | 1 | 4 | 2.000 |
| Gawler Ranges | 1 | 195 | 2 | 2 | 2 | 2 | 1 | 3 | 2.000 |
| Gibraltar Range |  | 307 | 4 | 2 | 3 | 2 | 1 | 3 | 2.500 |
| Gidgegannup |  | 206 | 3 | 2 | 2 | 3 | 1 | 4 | 2.500 |
| Gillingarra |  | 196 | 2 | 2 | 2 | 2 | 1 | 3 | 2.000 |
| Gippsland Lakes | 2 | 304 | 4 | 2 | 1 | 1 | 2 | 4 | 2.333 |
| Goonoo |  | 253 | 3 | 3 | 1 | 1 | 1 | 4 | 2.167 |
| Goose Island (Spencer Gulf) |  | 43 | 1 | 2 | 1 | 1 | 2 | 3 | 1.667 |
| Goyder Lagoon |  | 170 | 2 | 2 | 2 | 3 | 2 | 3 | 2.333 |
| Granite Downs |  | 131 | 2 | 2 | 2 | 3 | 1 | 3 | 2.167 |
| Great Sandy Strait |  | 362 | 4 | 1 | 2 | 1 | 2 | 4 | 2.333 |
| Greater Blue Mountains | 8 | 333 | 4 | 3 | 2 | 2 | 3 | 4 | 3.000 |
| Gregory National Park |  | 212 | 3 | 3 | 3 | 4 | 1 | 3 | 2.833 |
| Griffith Wetlands |  | 237 | 3 | 2 | 1 | 1 | 1 | 4 | 2.000 |
| Gulf Plains | 4 | 308 | 4 | 3 | 1 | 3 | 2 | 4 | 2.833 |
| Gulf St Vincent |  | 303 | 4 | 2 | 1 | 1 | 2 | 4 | 2.333 |
| Gum Lagoon |  | 251 | 3 | 2 | 1 | 1 | 1 | 3 | 1.833 |
| Gwydir Wetlands |  | 241 | 3 | 2 | 1 | 1 | 2 | 4 | 2.167 |
| Hastings-Macleay |  | 365 | 4 | 3 | 1 | 1 | 1 | 4 | 2.333 |
| Haul Round Island |  | 17 | 1 | 1 | 1 | 1 | 2 | 2 | 1.333 |
| Higginson Island |  | 17 | 1 | 1 | 1 | 1 | 2 | 4 | 1.667 |
| Hippolyte Rocks | 1 | 50 | 1 | 1 | 1 | 1 | 2 | 4 | 1.667 |
| Holleton |  | 169 | 2 | 2 | 1 | 2 | 1 | 2 | 1.667 |
| Houtman Abrolhos |  | 88 | 1 | 2 | 1 | 1 | 4 | 4 | 2.167 |
| Hunter Estuary | 3 | 356 | 4 | 2 | 1 | 1 | 2 | 4 | 2.333 |
| Hunter Island Group |  | 166 | 2 | 2 | 4 | 3 | 4 | 3 | 3.000 |
| Hunter Valley | 2 | 319 | 4 | 2 | 1 | 1 | 1 | 4 | 2.167 |
| Hyland Bay & Moyle Floodplain |  | 237 | 3 | 1 | 1 | 1 | 2 | 3 | 1.833 |
| Investigator Islands |  | 161 | 2 | 2 | 1 | 1 | 2 | 2 | 1.667 |
| Iron & McIlwraith Ranges | 7 | 310 | 4 | 2 | 3 | 2 | 1 | 2 | 2.333 |
| Islands North of Port Stewart |  | 217 | 3 | 1 | 1 | 1 | 4 | 2 | 2.000 |
| Islet off NE Grooyte Eylandt |  | 16 | 1 | 1 | 1 | 1 | 2 | 4 | 1.667 |
| Jalbarragup |  | 186 | 2 | 2 | 2 | 3 | 1 | 4 | 2.333 |
| Jervis Bay |  | 344 | 4 | 2 | 2 | 2 | 1 | 4 | 2.500 |
| Kakadu Savanna | 13 | 283 | 3 | 3 | 3 | 4 | 1 | 3 | 2.833 |
| Kangaroo Island | 3 | 236 | 3 | 4 | 2 | 2 | 2 | 3 | 2.667 |
| Karara & Lochada |  | 194 | 2 | 2 | 2 | 3 | 1 | 2 | 2.000 |
| Karroun Hill |  | 182 | 2 | 2 | 1 | 2 | 1 | 2 | 1.667 |
| Keep River | 2 | 236 | 3 | 3 | 2 | 4 | 1 | 4 | 2.833 |
| King Island |  | 176 | 2 | 3 | 4 | 4 | 4 | 4 | 3.500 |
| Koobabbie |  | 181 | 2 | 2 | 1 | 1 | 1 | 3 | 1.667 |
| Kwobrup-Badgebup |  | 177 | 2 | 2 | 3 | 1 | 1 | 4 | 2.167 |
| Lacepede Islands | 3 | 151 | 2 | 1 | 1 | 1 | 4 | 3 | 2.000 |
| Lake Argyle | 2 | 226 | 3 | 2 | 1 | 1 | 2 | 4 | 2.167 |
| Lake Barlee |  | 155 | 2 | 1 | 1 | 1 | 2 | 2 | 1.500 |
| Lake Bathurst |  | 257 | 3 | 2 | 1 | 1 | 2 | 4 | 2.167 |
| Lake Bindegolly |  | 206 | 3 | 2 | 1 | 3 | 2 | 3 | 2.333 |
| Lake Corangamite Complex |  | 257 | 3 | 2 | 1 | 1 | 2 | 4 | 2.167 |
| Lake Eyre |  | 179 | 2 | 1 | 1 | 1 | 2 | 2 | 1.500 |
| Lake Galilee |  | 194 | 2 | 1 | 1 | 1 | 2 | 4 | 1.833 |
| Lake Gore System |  | 173 | 2 | 2 | 1 | 1 | 2 | 4 | 2.000 |
| Lake Gregory/Paraku |  | 158 | 2 | 2 | 1 | 1 | 2 | 1 | 1.500 |
| Lake Hawdon System | 1 | 294 | 3 | 2 | 1 | 2 | 2 | 3 | 2.167 |
| Lake Machattie Area |  | 175 | 2 | 2 | 2 | 2 | 2 | 4 | 2.333 |
| Lake MacLeod |  | 186 | 2 | 2 | 2 | 1 | 2 | 3 | 2.000 |
| Lake Macquarie |  | 357 | 4 | 2 | 1 | 1 | 1 | 4 | 2.167 |
| Lake Magenta |  | 191 | 2 | 3 | 2 | 3 | 1 | 3 | 2.333 |
| Lake McLarty | 1 | 203 | 3 | 2 | 1 | 1 | 2 | 4 | 2.167 |
| Lake Newland |  | 219 | 3 | 2 | 1 | 1 | 2 | 2 | 1.833 |
| Lake Pleasant View System |  | 192 | 2 | 2 | 1 | 1 | 1 | 4 | 1.833 |
| Lake Sylvester System |  | 140 | 2 | 2 | 1 | 2 | 4 | 3 | 2.333 |
| Lake Torrens |  | 202 | 3 | 1 | 1 | 1 | 2 | 3 | 1.833 |
| Lake Warden System |  | 175 | 2 | 2 | 1 | 1 | 2 | 4 | 2.000 |
| Lake Wollumboola |  | 290 | 3 | 1 | 1 | 1 | 2 | 4 | 2.000 |
| Lake Woods |  | 159 | 2 | 1 | 1 | 1 | 2 | 2 | 1.500 |
| Lake Yamma Yamma |  | 177 | 2 | 2 | 1 | 1 | 2 | 3 | 1.833 |
| Lakes Alexandrina & Albert | 1 | 313 | 4 | 3 | 1 | 1 | 2 | 4 | 2.500 |
| Lakes Ballard & Marmion |  | 139 | 2 | 1 | 1 | 1 | 2 | 3 | 1.667 |
| Lakes Muncoonie, Mumbleberry & Torquinie |  | 166 | 2 | 1 | 2 | 2 | 2 | 4 | 2.167 |
| Lawrence Rocks |  | 49 | 1 | 1 | 1 | 1 | 3 | 4 | 1.833 |
| Legune (Joseph Bonaparte Bay) |  | 253 | 3 | 1 | 1 | 1 | 2 | 2 | 1.667 |
| Lilyvale |  | 255 | 3 | 2 | 1 | 2 | 1 | 2 | 1.833 |
| Limmen Bight |  | 228 | 3 | 1 | 2 | 1 | 2 | 1 | 1.667 |
| Little Desert | 6 | 259 | 3 | 2 | 1 | 1 | 1 | 2 | 1.667 |
| Lockerbie Scrub | 2 | 259 | 3 | 1 | 2 | 1 | 3 | 1 | 1.833 |
| Lord Howe Island | 1 | 97 | 1 | 2 | 1 | 1 | 4 | 4 | 2.167 |
| Low Rocks & Sterna Island (Kimberley) |  | 13 | 1 | 1 | 1 | 1 | 2 | 2 | 1.333 |
| Lowbidgee Floodplain | 1 | 239 | 3 | 1 | 1 | 1 | 3 | 3 | 2.000 |
| Lowendal Islands |  | 89 | 1 | 1 | 1 | 1 | 2 | 2 | 1.333 |
| Lower Brodribb River |  | 242 | 3 | 2 | 1 | 1 | 1 | 4 | 2.000 |
| Maatsuyker Island Group |  | 143 | 2 | 1 | 1 | 1 | 4 | 3 | 2.000 |
| Macquarie Island |  | 91 | 1 | 4 | 1 | 1 | 3 | 1 | 1.833 |
| Macquarie Marshes |  | 237 | 3 | 2 | 1 | 1 | 2 | 4 | 2.167 |
| Mandora Marsh & Anna Plains |  | 221 | 3 | 2 | 2 | 1 | 2 | 2 | 2.000 |
| Manowar & Rocky Islands |  | 16 | 1 | 1 | 1 | 1 | 3 | 4 | 1.833 |
| Maria Island |  | 169 | 2 | 3 | 4 | 4 | 1 | 4 | 3.000 |
| Marion Bay |  | 211 | 3 | 2 | 1 | 1 | 2 | 4 | 2.167 |
| Maryborough-Dunolly Box-Ironbark Region | 2 | 268 | 3 | 2 | 1 | 1 | 1 | 3 | 1.833 |
| Melaleuca to Birchs Inlet | 1 | 186 | 2 | 2 | 4 | 4 | 2 | 3 | 2.833 |
| Menindee Lakes |  | 205 | 3 | 1 | 1 | 1 | 2 | 3 | 1.833 |
| Mewstone |  | 49 | 1 | 2 | 1 | 1 | 3 | 2 | 1.667 |
| Michaelmas Cay | 6 | 20 | 1 | 1 | 1 | 1 | 2 | 4 | 1.667 |
| Milingimbi Islands |  | 163 | 2 | 1 | 1 | 1 | 2 | 2 | 1.500 |
| Montebello Islands |  | 89 | 1 | 2 | 1 | 1 | 2 | 2 | 1.500 |
| Moora |  | 192 | 2 | 2 | 1 | 1 | 1 | 4 | 1.833 |
| Morehead River | 6 | 239 | 3 | 3 | 2 | 3 | 1 | 2 | 2.333 |
| Moreton Bay & Pumicestone Passage | 1 | 389 | 4 | 2 | 2 | 1 | 2 | 4 | 2.500 |
| Mornington Sanctuary | 1 | 189 | 2 | 4 | 2 | 4 | 1 | 2 | 2.500 |
| Moulting Lagoon |  | 205 | 3 | 1 | 1 | 1 | 2 | 3 | 1.833 |
| Mount Gibson & Charles Darwin |  | 191 | 2 | 2 | 2 | 4 | 1 | 2 | 2.167 |
| Mount Lyndhurst | 6 | 172 | 2 | 2 | 2 | 3 | 1 | 4 | 2.333 |
| Mudgee-Wollar | 1 | 287 | 3 | 2 | 2 | 2 | 1 | 4 | 2.333 |
| Muir-Unicup Wetlands | 3 | 185 | 2 | 2 | 1 | 1 | 1 | 3 | 1.667 |
| Mundaring-Kalamunda |  | 214 | 3 | 2 | 3 | 3 | 1 | 4 | 2.667 |
| Murray-Sunset, Hattah & Annuello | 8 | 271 | 3 | 3 | 1 | 2 | 1 | 4 | 2.333 |
| Murrumbidgee Red Gums |  | 248 | 3 | 2 | 2 | 1 | 1 | 4 | 2.167 |
| Nadgee to Mallacoota Inlet | 2 | 306 | 4 | 2 | 2 | 2 | 1 | 4 | 2.500 |
| Narran Wetlands |  | 210 | 3 | 2 | 1 | 1 | 2 | 4 | 2.167 |
| Natimuk-Douglas Wetlands |  | 260 | 3 | 1 | 1 | 1 | 2 | 3 | 1.833 |
| New England |  | 310 | 4 | 2 | 3 | 2 | 1 | 4 | 2.667 |
| Night Island (Bass Strait) |  | 46 | 1 | 1 | 1 | 1 | 2 | 3 | 1.500 |
| Nightcap Range |  | 328 | 4 | 2 | 3 | 2 | 1 | 4 | 2.667 |
| Ninth & Little Waterhouse Islands |  | 49 | 1 | 1 | 1 | 1 | 2 | 4 | 1.667 |
| North Dandalup |  | 209 | 3 | 2 | 2 | 3 | 1 | 4 | 2.500 |
| North Victorian Wetlands | 1 | 266 | 3 | 2 | 1 | 1 | 2 | 2 | 1.833 |
| Northern Swan Coastal Plain | 3 | 264 | 3 | 2 | 2 | 3 | 1 | 4 | 2.500 |
| North-west Tasmanian Coast | 2 | 214 | 3 | 3 | 3 | 4 | 2 | 3 | 3.000 |
| Nuyts Archipelago |  | 154 | 2 | 1 | 1 | 1 | 4 | 2 | 1.833 |
| Ord Irrigation Area | 3 | 241 | 3 | 3 | 2 | 3 | 1 | 4 | 2.667 |
| Orford (Tasmania) |  | 205 | 3 | 2 | 1 | 1 | 2 | 4 | 2.167 |
| Otway Range | 5 | 305 | 4 | 1 | 2 | 2 | 1 | 4 | 2.333 |
| Owingup Swamp & Boat Harbour Wetlands |  | 194 | 2 | 2 | 1 | 1 | 1 | 4 | 1.833 |
| Palmgrove |  | 234 | 3 | 2 | 2 | 2 | 1 | 4 | 2.333 |
| Paluma |  | 343 | 4 | 2 | 3 | 3 | 1 | 4 | 2.833 |
| Paroo Floodplain & Currawinya | 4 | 228 | 3 | 2 | 1 | 3 | 2 | 1 | 2.000 |
| Patho Plains | 2 | 266 | 3 | 2 | 1 | 1 | 1 | 4 | 2.000 |
| Pearce, Urquhart & Hervey Islands (Sir Edward Pellew Group) |  | 16 | 1 | 1 | 1 | 1 | 2 | 1 | 1.167 |
| Pedra Branca |  | 49 | 1 | 2 | 1 | 1 | 3 | 3 | 1.833 |
| Peebinga |  | 241 | 3 | 2 | 1 | 1 | 1 | 3 | 1.833 |
| Peel-Harvey Estuary | 1 | 240 | 3 | 2 | 1 | 1 | 2 | 4 | 2.167 |
| Phillip Island | 4 | 233 | 3 | 2 | 1 | 1 | 4 | 4 | 2.500 |
| Pilliga |  | 259 | 3 | 2 | 1 | 1 | 1 | 3 | 1.833 |
| Pink Lake (Esperance) | 1 | 174 | 2 | 2 | 1 | 1 | 2 | 4 | 2.000 |
| Piper Islands |  | 21 | 1 | 1 | 1 | 1 | 4 | 3 | 1.833 |
| Port Davey Islands |  | 175 | 2 | 1 | 1 | 1 | 4 | 3 | 2.000 |
| Port Fairy to Warrnambool | 1 | 285 | 3 | 2 | 1 | 1 | 1 | 4 | 2.000 |
| Port Hedland Saltworks |  | 187 | 2 | 1 | 2 | 1 | 2 | 4 | 2.000 |
| Port McArthur Tidal Wetlands System |  | 229 | 3 | 1 | 2 | 1 | 2 | 1 | 1.667 |
| Prince Regent & Mitchell River | 4 | 255 | 3 | 3 | 3 | 4 | 1 | 2 | 2.667 |
| Puckapunyal | 1 | 268 | 3 | 3 | 1 | 1 | 1 | 4 | 2.167 |
| Quoin Bluff & Freycinet Island (Shark Bay) |  | 132 | 2 | 1 | 1 | 1 | 2 | 1 | 1.333 |
| Raine Island, Moulter & Maclennan Cays |  | 20 | 1 | 1 | 1 | 1 | 4 | 1 | 1.500 |
| Recherche Archipelago | 1 | 223 | 3 | 2 | 2 | 2 | 4 | 3 | 2.667 |
| Repulse Bay to Ince Bay |  | 329 | 4 | 2 | 2 | 1 | 2 | 4 | 2.500 |
| Richmond Woodlands | 3 | 302 | 4 | 2 | 1 | 1 | 1 | 4 | 2.167 |
| Riverina Plains | 3 | 264 | 3 | 2 | 1 | 1 | 1 | 3 | 1.833 |
| Riverland Mallee | 9 | 258 | 3 | 3 | 2 | 2 | 1 | 3 | 2.333 |
| Robbins Passage & Boullanger Bay |  | 205 | 3 | 3 | 3 | 4 | 4 | 3 | 3.333 |
| Roebuck Bay | 4 | 256 | 3 | 3 | 2 | 1 | 2 | 4 | 2.500 |
| Rottnest Island | 1 | 171 | 2 | 2 | 1 | 1 | 2 | 4 | 2.000 |
| Rubicon Estuary |  | 199 | 2 | 1 | 1 | 1 | 2 | 4 | 1.833 |
| Rushworth Box-Ironbark Region | 1 | 265 | 3 | 3 | 1 | 1 | 1 | 4 | 2.167 |
| Sandy Island & Low Rock (Gulf of Carpentaria) |  | 16 | 1 | 1 | 1 | 1 | 2 | 4 | 1.667 |
| Sandy Island (Windy Harbour) |  | 40 | 1 | 2 | 1 | 1 | 4 | 4 | 2.167 |
| Scenic Rim | 4 | 350 | 4 | 3 | 3 | 3 | 1 | 4 | 3.000 |
| Seagull Island (Tiwi Islands) |  | 159 | 2 | 1 | 1 | 1 | 2 | 3 | 1.667 |
| Seagull Lake (Eyre Peninsula) |  | 180 | 2 | 2 | 1 | 1 | 2 | 4 | 2.000 |
| Shag Reef (Bass Strait) |  | 46 | 1 | 1 | 1 | 1 | 2 | 2 | 1.333 |
| Shallow Inlet | 1 | 283 | 3 | 2 | 1 | 1 | 2 | 4 | 2.167 |
| Shoal Bay (Darwin) |  | 264 | 3 | 1 | 1 | 1 | 2 | 4 | 2.000 |
| Shoalwater Bay (Rockhampton) |  | 317 | 4 | 2 | 2 | 1 | 2 | 3 | 2.333 |
| Simpson Desert |  | 194 | 2 | 2 | 2 | 4 | 1 | 3 | 2.333 |
| Sir Joseph Banks Islands |  | 155 | 2 | 2 | 1 | 1 | 4 | 2 | 2.000 |
| South Arm | 1 | 211 | 3 | 1 | 1 | 1 | 2 | 4 | 2.000 |
| South Barnard Islands |  | 20 | 1 | 1 | 1 | 1 | 2 | 4 | 1.667 |
| South-east Tasmania | 6 | 225 | 3 | 3 | 3 | 4 | 1 | 4 | 3.000 |
| Southern NSW Mallee | 2 | 261 | 3 | 2 | 1 | 1 | 1 | 4 | 2.000 |
| Southern Yorke Peninsula |  | 261 | 3 | 3 | 1 | 2 | 1 | 3 | 2.167 |
| South-west Slopes of NSW |  | 309 | 4 | 3 | 2 | 1 | 1 | 3 | 2.333 |
| Spencer Gulf |  | 276 | 3 | 1 | 1 | 1 | 2 | 4 | 2.000 |
| St Arnaud Box-Ironbark Region | 3 | 260 | 3 | 3 | 1 | 1 | 1 | 3 | 2.000 |
| St Helens (Tasmania) |  | 202 | 3 | 2 | 1 | 1 | 4 | 3 | 2.333 |
| Staaten River |  | 201 | 3 | 3 | 2 | 3 | 1 | 2 | 2.333 |
| Stapleton Island |  | 21 | 2 | 1 | 1 | 1 | 4 | 2 | 1.833 |
| Stirling Range | 7 | 202 | 3 | 3 | 2 | 4 | 1 | 4 | 2.833 |
| Strzelecki Desert Lakes | 6 | 182 | 2 | 1 | 2 | 2 | 2 | 3 | 2.000 |
| Sudbury Reef |  | 20 | 1 | 1 | 1 | 1 | 2 | 4 | 1.667 |
| Sunday Island (Exmouth Gulf) |  | 17 | 1 | 1 | 1 | 1 | 2 | 2 | 1.333 |
| Swain Reefs |  | 28 | 1 | 1 | 1 | 1 | 2 | 2 | 1.333 |
| Swan Bay & Port Phillip Bay Islands |  | 301 | 4 | 3 | 1 | 1 | 2 | 4 | 2.500 |
| Tamar Wetlands |  | 202 | 3 | 1 | 1 | 1 | 2 | 4 | 2.000 |
| Tamborine Mountain |  | 330 | 4 | 2 | 2 | 2 | 1 | 4 | 2.500 |
| Tarrabool Lake-Eva Downs Swamp System |  | 145 | 2 | 1 | 1 | 1 | 2 | 3 | 1.667 |
| Tasman Island |  | 164 | 2 | 1 | 1 | 1 | 3 | 4 | 2.000 |
| The Lakes (Western Australia) |  | 195 | 2 | 2 | 2 | 3 | 1 | 4 | 2.333 |
| Three Hummocks Island (Arnhem Land) |  | 18 | 1 | 1 | 1 | 1 | 2 | 4 | 1.667 |
| Three Sisters (Bass Strait) |  | 45 | 1 | 1 | 1 | 1 | 2 | 4 | 1.667 |
| Tiwi Islands |  | 189 | 2 | 3 | 2 | 3 | 2 | 4 | 2.667 |
| Tourville & Murat Bays |  | 204 | 3 | 1 | 1 | 1 | 2 | 4 | 2.000 |
| Towerrining Lake & Moodiarrup Swamps |  | 169 | 2 | 2 | 1 | 1 | 2 | 4 | 2.000 |
| Traprock |  | 276 | 3 | 2 | 1 | 1 | 1 | 4 | 2.000 |
| Troubridge Island |  | 42 | 1 | 2 | 1 | 1 | 2 | 4 | 1.833 |
| Tuggerah |  | 354 | 4 | 2 | 1 | 1 | 2 | 4 | 2.333 |
| Two Peoples Bay & Mount Manypeaks | 6 | 238 | 3 | 4 | 3 | 4 | 3 | 4 | 3.500 |
| Ulladulla to Merimbula |  | 340 | 4 | 2 | 1 | 2 | 1 | 3 | 2.167 |
| Venus Bay |  | 225 | 3 | 2 | 1 | 1 | 2 | 2 | 1.833 |
| Walebing |  | 184 | 2 | 2 | 2 | 2 | 1 | 3 | 2.000 |
| Wandown |  | 243 | 3 | 2 | 1 | 1 | 1 | 3 | 1.833 |
| Warby-Chiltern Box-Ironbark Region | 4 | 270 | 3 | 4 | 1 | 1 | 1 | 3 | 2.167 |
| Watervalley Wetlands |  | 249 | 3 | 2 | 1 | 1 | 2 | 3 | 2.000 |
| Wedge Island |  | 159 | 2 | 1 | 1 | 1 | 3 | 4 | 2.000 |
| Werribee & Avalon | 7 | 311 | 4 | 2 | 1 | 1 | 2 | 4 | 2.333 |
| Werrikimbe |  | 303 | 4 | 2 | 2 | 2 | 1 | 4 | 2.500 |
| Western Port | 1 | 305 | 4 | 2 | 1 | 1 | 2 | 4 | 2.333 |
| Wilson Reef (Great Barrier Reef) |  | 20 | 1 | 1 | 1 | 1 | 3 | 4 | 1.833 |
| Wilsons Promontory Islands |  | 280 | 3 | 1 | 1 | 1 | 4 | 4 | 2.333 |
| Wollogorang |  | 192 | 2 | 2 | 2 | 2 | 1 | 3 | 2.000 |
| Wooroonooran | 16 | 369 | 4 | 2 | 3 | 4 | 1 | 4 | 3.000 |
| Wyndham | 1 | 240 | 3 | 2 | 2 | 3 | 1 | 4 | 2.500 |
| Wyperfeld, Big Desert & Ngarkat | 6 | 280 | 3 | 3 | 2 | 2 | 1 | 3 | 2.333 |
| Yalgorup |  | 207 | 3 | 2 | 1 | 1 | 2 | 4 | 2.167 |
| Yambuk |  | 288 | 3 | 3 | 1 | 1 | 1 | 4 | 2.167 |
| Yinberrie Hills | 4 | 210 | 3 | 3 | 2 | 4 | 1 | 2 | 2.500 |
| Averages | 3.61 | 204 | 2.548 | 1.890 | 1.523 | 1.658 | 1.874 | 3.223 | 2.119 |
